# Application of biological agents in the treatment of anti-neutrophil cytoplasmic antibody-associated vasculitis

**DOI:** 10.3389/fphar.2024.1378384

**Published:** 2024-05-20

**Authors:** Weijun Liu, Guanyuan Tian, Chao Chen, Mingying Zhang, Zhanmao Chen, Tietao Chen, Zhibin Lin, Wuzhong Wu, Yiqaing Wu, Kefei Wu, Qinghua Liu

**Affiliations:** ^1^ Department of Nephrology, Jieyang People’s Hospital, Jieyang, China; ^2^ Department of Nephrology, The First Affiliated Hospital, Sun Yat-sen University, Guangzhou, China; ^3^ NHC Key Laboratory of Clinical Nephrology (Sun Yat-sen University) and Guangdong Provincial Key Laboratory of Nephrology, Guangzhou, China

**Keywords:** anti-neutrophil cytoplasmic antibody, biological agents, vasculitis, treatment, microscopic polyangiitis, granulomatosis with polyangiitis, rituximab

## Abstract

Anti-neutrophil cytoplasmic antibody (ANCA)-associated vasculitis (AAV) has been traditionally treated using glucocorticoids and immunosuppressants. However, these treatment modes are associated with high recurrence AAV rates and adverse reactions. Therefore, treatment strategies for AAV need to be urgently optimized. The efficacy and safety of biological agents in the treatment of vasculitis have been clinically validated. This review comprehensively summarizes the evidence-based support for the clinical use of existing biological agents in AAV. The findings reveal that multiple biological agents not only effectively reduce the adverse reactions associated with glucocorticoids and immunosuppressants but also demonstrate significant therapeutic efficacy. Notably, rituximab, an anti-CD20 antibody, has emerged as a first-line treatment option for AAV. Mepolizumab has shown promising results in relapsed and refractory eosinophilic granulomatosis with polyangiitis. Other biological agents targeting cytokines, complement, and other pathways have also demonstrated clinical benefits in recent studies. The widespread application of biological agents provides new insights into the treatment of AAV and is expected to drive further clinical research. These advancements not only improve patient outcomes but also offer more possibilities and hope in the field of AAV treatment.

## 1 Introduction

Anti-neutrophil cytoplasmic antibody (ANCA)-associated vasculitis (AAV) is a group of systemic diseases, which are characterized by the ANCA-mediated inflammation and necrosis of small blood vessel walls. The clinical types of AAV include microscopic polyangiitis (MPA), granulomatosis with polyangiitis (GPA), and eosinophilic granulomatosis with polyangiitis (EGPA). Proteinase 3 (PR3)-ANCA and myeloperoxidase (MPO)-ANCA are the two main ANCA antigens, which are found in the cytoplasm of neutrophils. PR3-ANCA is usually associated with GPA, whereas MPO-ANCA is primarily found in the context of MPA (Bossuyt et al., 2017). AAV can occur in all age groups, with a peak incidence in individuals aged 50–60 years. The annual incidence of AAV is reported to be 1.2–2.0 cases per 100,000 people, while its prevalence is 4.6–18.4 cases per 100,000 people (Watts et al., 2015). AVV is traditionally treated with immunosuppressive agents such as glucocorticoids and cyclophosphamide (CYC). However, long-term use of these agents can lead to various treatment-related complications. For instance, glucocorticoid use may cause infections (Cutolo et al., 2008), osteoporosis (Chotiyarnwong and McCloskey, 2020), impaired glucose tolerance (Hwang and Weiss, 2014), and other side effects. Meanwhile, the metabolites of CYC induce bladder and reproductive organ toxicity, which may lead to complications such as the formation of malignant tumors (Rahmattulla et al., 2015) and infertility (Feng et al., 1972) in the long term. Although immunotherapy has somewhat improved the outcomes of patients with AAV, disease recurrence is still very common. Indeed, 30%–50% of AAV patients relapse within 5 years of disease onset, usually within 12–18 months after stopping immunosuppressant therapy (Geetha and Jefferson, 2020). Therefore, treatment strategies for AAV need to be urgently optimized. Biological agents (biologics) target pathogenic mechanisms with a high degree of specificity to rapidly relieve symptoms, lower relapse rates, permit a reduction of corticosteroid dosage, and improve AAV patient quality of life. To date, numerous biologics have shown promise in the treatment of AAV; this review summarizes their clinical applications.

## 2 Biologics

**FIGURE 1:**
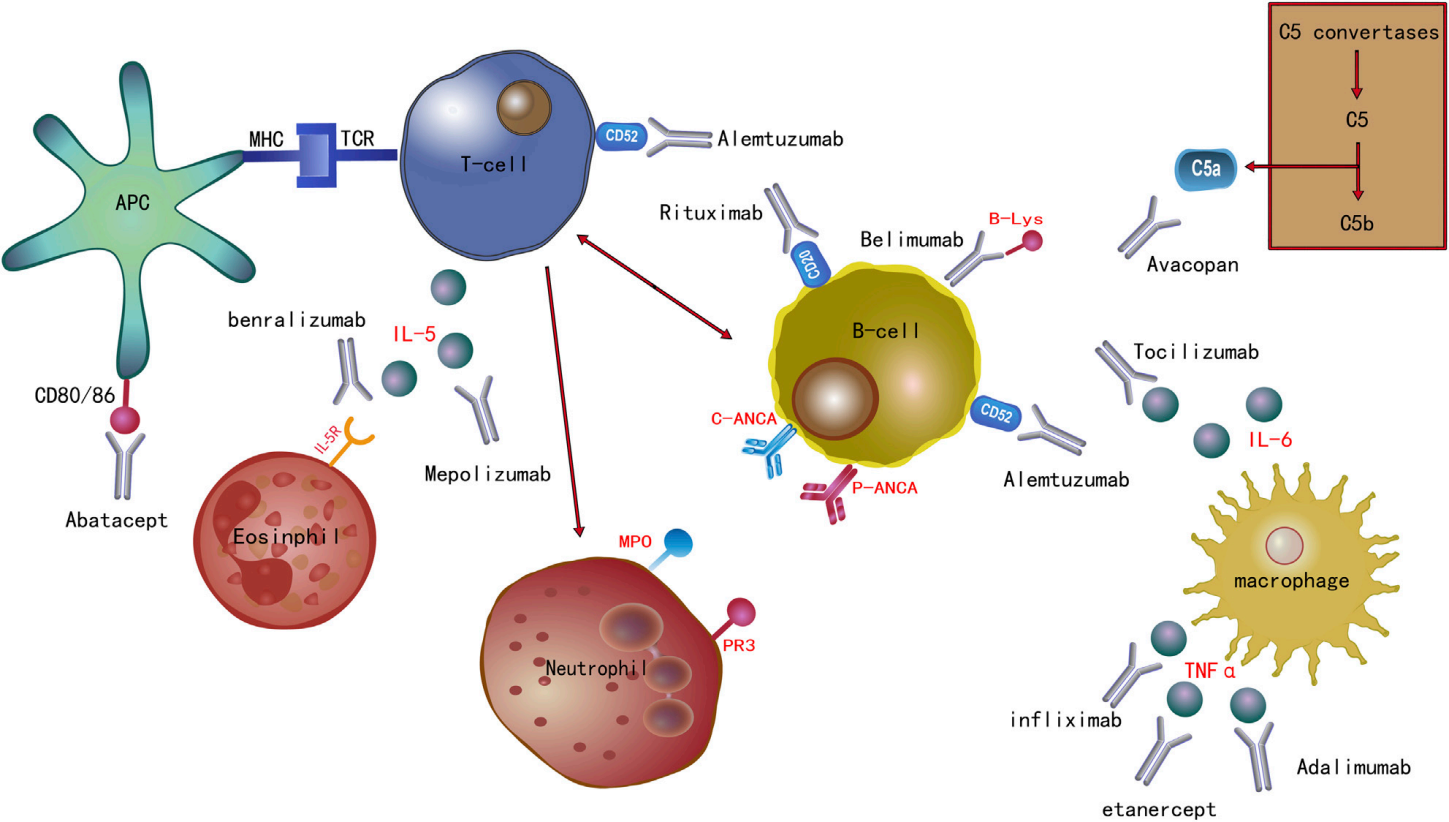
Biological agents used to treat AAV and their related molecular targets.

In this section of the review, we have visually presented the target sites of the current biological agents used in the treatment of AAV through illustrative images. Furthermore, we have also delved into the progress of the clinical application of these medications. (Figure 1).

Biological agents currently used in the treatment of anti-neutrophil cytoplasmic antibody-associated vasculitis and their associated molecular targets. Rituximab is an anti-CD20 antibody. Alemtuzumab is an anti-CD52 antibody. Abatacept targets CD80/86. Infliximab, etanercept, and adalimumab target the cytokine tumor necrosis factor (TNF)-α. Interleukin (IL)-6 and IL-5 are the targets of tocilizumab and mepolizumab, respectively. Benralizumab targets IL-5R. Avacopan targets complement factor C5a. The B lymphocyte stimulator (B-Lys) is the target of belimumab.

### 2.1 Anti-CD20 monoclonal antibody

A strong correlation between the presence of ANCA in serum and AAV pathophysiology suggests a pathogenic role for humoral immunity in AAV (Wilde et al., 2011). CD20 is a protein present on the surface of mature B cells, which supports the strategy of using anti-CD20 antibodies to treat AAV patients.

Rituximab (RTX) is an anti-CD20 IgG1 chimeric mouse/human monoclonal antibody, which was approved by the US Food and Drug Administration (FDA) in 2011 for the treatment of AAV. Its efficacy has been fully affirmed. A UK-based 2020 expert consensus (Tieu et al., 2020) recommended the prolonged use of RTX (i.e., 500 mg or 1,000 mg every 6 months for 2 years) to maintain AAV remission. If the risk of recurrence remained high after 2 years of maintenance therapy, it is recommended that the treatment period is extended to 5 years. Discontinuation of disease-modifying anti-rheumatic drugs is recommended after RTX initiation, whereas hormonal reduction should be completely discontinued within 6–12 months. The 2021 Kidney Disease Improving Global Outcomes (KDIGO) guidelines (Kidney Disease: Improving Global Outcomes KDIGO Glomerular Diseases Work Group, 2021) recommend administering glucocorticoids in combination with CYC or RTX as an initial treatment regimen in *de novo* AAV. For patients with serum creatinine levels > 354 μmol/L, the combination of CYC and glucocorticoids is the first choice induction therapy, although the combination of RTX and CYC can also be considered. For maintenance therapy after induced remission, azathioprine (AZA) or RTX combined with low-dose corticosteroids or RTX alone is recommended. RTX re-induction therapy is preferred for relapsed patients. For patients with refractory diseases, the amount of glucocorticoid can be increased, and RTX or CYC should be included in the regimen. These viewpoints are mainly derived from randomized clinical trials (RCTs) such as RITUXVAS (Jones et al., 2015; 2010a), RAVE (Stone et al., 2010), MAINRITSAN (Guillevin et al., 2014; Charles et al., 2020, 2018; Terrier et al., 2018), RITAZAREM (Gopaluni et al., 2017; Smith et al., 2020, 2023), and will not be discussed further here. The 2022 European League Against Rheumatism (EULAR) guidelines (Hellmich et al., 2024) and the 2023 Pan American League of Associations for Rheumatology (PANLAR) guidelines (Magri et al., 2023) similarly recommend glucocorticoid use in combination with RTX for the induction of remission in patients with new-onset or relapsing GPA or MPA. Moreover, RTX should be indicated for patients with disease recurrence. Over the past 2 years, new insights into AAV treatment have been revealed. In severely ill patients, for instance, there does not appear to be a clear difference between the efficacies of RTX and CYC. A multicenter retrospective study (Morel et al., 2022) involving 153 patients with AAV, who had serum creatinine levels ≥ 350 μmol, assessed the induction of remission with either RTX or CYC combined hormone therapy; however, no significant difference was observed between the efficacies of the two regimens. Studies (Roccatello et al., 2022) by Roccatello et al. have drawn similar conclusions. The most recent real-world study (Ishikawa et al., 2023), which included 687 patients with life-threatening AAV, who presented with rapidly progressive glomerulonephritis and/or alveolar hemorrhage, also suggested that RTX and CYC had similar short-term effects on mortality. Another study (Morel et al., 2022) suggested that compared with RTX, CYC-assisted plasmapheresis had a higher dialysis-free rate at 12 months. Meanwhile, RTX use may be associated with a lower risk of fungal infection and pneumocystis pneumonia, but a higher risk of short-term renal function deterioration, which may require hemodialysis maintenance (Ishikawa et al., 2023). In a retrospective study (Sorin et al., 2022) involving 17 patients with refractory GPA treated with the RTX/methotrexate (MTX) combination, 94% achieved overall remission, indicating that this combination can be used as a salvage therapy with an acceptable safety profile. The MAINRITSAN trial (Delestre et al., 2023) compared the outcomes of 277 AAV patients who were treated with AZA or an 18-month regimen of tailored RTX. During treatment, 500 mg RTX was reinfused based on testing every 3 months if peripheral CD19^+^ B cells or ANCA reappeared or ANCA titers increased. The results showed that the 84-month response rate was higher in the group receiving the 18-month fixed RTX regimen (500 mg of RTX at days 0 and 14 and at months 6, 12, and 18) than in the one undergoing AZA treatment. In addition, extending RTX treatment to 36 months did not reduce the long-term AAV recurrence rate compared with that of the group receiving the 18-month fixed RTX regimen. A multicenter retrospective study (Machet et al., 2023), which included 116 patients with AAV, showed that 12% of patients receiving RTX induction therapy were RTX-resistant at 3 months. These patients often have localized disease and rarely receive the initial methylprednisolone pulse and prophylactic trimethoprim-sulfamethoxazole therapy. The LoVAS study (Furuta et al., 2021; Furuta et al., 2023), which included 134 patients with non-severe AAV, compared the performance of RTX combined with low-dose or high-dose glucocorticoid induction regimens. The results showed that a reduced-dose glucocorticoid plus rituximab regimen was noninferior to a high-dose glucocorticoid plus rituximab regimen. At 24 months of follow-up, the incidence of recurrence, death, and end-stage renal disease were not significantly different between the low-dose and the high-dose glucocorticoid groups. Given that glucocorticoids were discontinued at 5 months in the low-dose glucocorticoid group, these results may imply that the continued administration of low-dose glucocorticoid therapy during remission maintenance with RTX is not necessary to prevent relapse in patients with AAV.

In terms of safety assessment, both the KDIGO guidelines (, 2024) and multiple authoritative studies (Jones et al., 2010a; Stone et al., 2010; Gérard et al., 2023) have indicated that the incidence of infections is roughly comparable between RTX and CYC when used as first-line induction remission therapies. However, there are differing views within the academic community regarding the safety comparison between these two treatment options. A study Jones et al. (2015) by compared the safety of two treatment combinations: RTX induction therapy followed by AZA maintenance therapy (RTX/AZA) and CYC induction therapy followed by AZA maintenance therapy (CYC/AZA). The primary outcomes observed were death, end-stage renal disease, and recurrence at 24 months. The results showed that the incidence rate in the RTX/AZA group was 42%, while that in the CYC/AZA group was 36%, with no significant difference found between the two. Nevertheless, another multicenter real-world study (Thomas et al., 2021) suggested that CYC induction therapy may lead to a higher incidence of severe infections compared to RTX. Additionally, a comprehensive meta-analysis (Vassilopoulos et al., 2023) found that the cumulative incidence of severe infections was significantly higher in the CYC/AZA group compared to the RTX/AZA group during the total follow-up period. It is worth mentioning that the 2023 RAVE study (Odler et al., 2023) provided new evidence. The study included data from 197 patients and showed that the RTX/AZA group had a similar incidence of severe infections compared to the CYC/AZA group. However, it is noteworthy that the onset of infections occurred earlier in the CYC/AZA group, with 82% of severe infections occurring within the first 6 months after the start of the trial. The study also found that a higher baseline CD19^+^ B-cell count and the use of trimethoprim-sulfamethoxazole for prevention could reduce the risk of severe infections such as Pneumocystis jirovecii. A study (Liu et al., 2023) focusing on Chinese patients with AAV found that low-dose RTX therapy (total dose of 400 mg within 4 weeks) was comparable in efficacy to CYC therapy but significantly reduced the incidence of serious adverse events (SAEs). Similarly, a real-world study (Ishikawa et al., 2024) in Japan suggested that RTX may have a lower risk of fungal infections and Pneumocystis pneumonia compared to CYC in critically ill patients with AAV. It is important to note that hypogammaglobulinemia is commonly observed in AAV patients receiving RTX therapy, which may be related to patient age and cumulative doses of glucocorticoids. This condition may increase the risk of infections, but immunoglobulin replacement therapy can mitigate this risk (Shah et al., 2017; Tieu et al., 2021; Podestà et al., 2023; Smith et al., 2023). During the maintenance therapy phase, the RITAZAREM study (Jones et al., 2010b) found no significant difference in infection rates between the RTX and AZA groups, despite a lower incidence of SAEs in the RTX group. However, a comprehensive meta-analysis (Lee and Song, 2022) comparing the efficacy and safety of various maintenance therapies showed that RTX performed best in reducing recurrence rates, while MMF had the lowest rate of severe infections. Another study (Thomas et al., 2021) indicated that during maintenance therapy, the incidence of severe infections was similar among RTX and other drugs. Additionally, the LoVAS study (Furuta et al., 2023) explored the safety of RTX combined with different doses of glucocorticoids. The results showed that during the 24-month follow-up period, the incidence of SAEs and infections was significantly lower in the low-dose group compared to the high-dose group. Furthermore, the long-term follow-up results of up to 4 years from the RaVeR study (Merkel et al., 2021) confirmed that the safety of long-term RTX treatment for AAV patients is consistent with short-term treatment and comparable to its safety profile in other autoimmune diseases. In conclusion, while RTX and CYC have similar infection rates during the induction phase of remission, RTX may demonstrate a lower risk of infections in certain specific situations. In the maintenance phase, RTX has shown significant efficacy in reducing recurrence rates, and the safety of long-term RTX use has been supported by research. However, clinicians should closely monitor and appropriately manage low gammaglobulinemia and its associated infection risk in patients receiving RTX therapy.

For specific patient populations, the KDIGO guidelines (, 2024) suggest that the use of RTX is superior to CYC for induction therapy in frail elderly patients, although the specific evidence remains unclear. Meta-analyses (Morris et al., 2020) have shown that in patients with AAV aged 75 and older, the use of CYC or RTX for induction therapy is significantly associated with a lower risk of 2-year mortality. Another multicenter retrospective survival analysis (Aqeel et al., 2023) indicates that CYC, CYC + RTX, and RTX alone are equally effective in inducing remission in AAV patients over the age of 60. Compared to treatment regimens containing CYC, the use of RTX alone may reduce the risk of bone marrow suppression. Research by Weiner et al. (2020) has demonstrated that both CYC and RTX therapies can reduce permanent organ damage in elderly patients with AAV. However, the use of higher doses of glucocorticoids within the first 3 months is associated with treatment-related injuries and fatal infections. A multicenter cohort study (Thietart et al., 2022) examined the outcomes and adverse events of RTX therapy in AAV patients aged 75 and older and found that most patients achieved sustained remission without recurrence. When RTX is used as induction therapy in combination with high-dose glucocorticoid regimens, the incidence of severe infections and death is higher; however, when used as maintenance therapy, these rates are not as high. Nevertheless, a retrospective study Timlin et al. (2015) by in AAV patients aged 60 and older found that while RTX is effective in inducing remission, the incidence of infections is higher. Prospective survival analysis (McGovern et al., 2020) suggests that age, frailty score, and CRP levels at the time of presentation are independently associated with mortality in elderly AAV patients. Although the CYC induction therapy group showed higher survival rates compared to the RTX group, this may be due to the younger age of patients in the CYC group, and further studies are needed to validate this observation. In conclusion, there is still a lack of high-quality research in this field, such as RCTs. Based on the limited evidence available, RTX therapy can be considered a priority choice for elderly patients. However, when RTX is contraindicated or the patient’s economic situation is poor, the CYC regimen is also a reasonable option. When using these treatment regimens, attention should be paid to preventing the occurrence of infections.

### 2.2 B cell activating factor inhibitors

The lymphocyte stimulating factor (BLyS) is a new member of the tumor necrosis factor (TNF) family, which has a unique role in B cell development/differentiation and autoimmune disease (Do and Chen-Kiang, 2002). BLyS is expressed by neutrophils, which are key cells in the pathogenesis of AAV, as elevated concentrations of circulating BLyS are reported in patients with AAV (Holden et al., 2011; Carter et al., 2013).

Belimumab, a human IgG1λ monoclonal antibody against BLyS, has been approved for the treatment of active, autoantibody-associated systemic lupus erythematosus in adults receiving standard therapy. In an RCT (Jayne et al., 2019), patients with AAV were randomized in a 1:1 ratio to receive AZA (2 mg/kg/day), low-dose oral glucocorticoids (≤10 mg/day), and intravenous belimumab (10 mg/kg) after induction of remission with RTX or CYC plus glucocorticoids. The control group received the placebo. The results suggested that the risk of AAV recurrence was not reduced in the experimental group versus the placebo group; however, no recurrence (0/14) was seen in patients who received RTX induction therapy followed by belimumab maintenance therapy. By contrast, three (23.1%) of the 13 patients who achieved disease remission with RTX in the placebo group relapsed. These findings suggest that dual, B-cell-targeted immunotherapy (i.e., B cell depletion + BLyS blockade) may be more effective than either treatment alone. Notably, however, the study in question had a small sample size and contained some errors. The most recent COMBIVAS study (McClure et al., 2023) included a randomized, double-blind, placebo-controlled trial investigating the mechanisms of sequential treatment of GPA with RTX and belimumab. The primary end point of the trial was the time taken for conversion of PR3-ANCA to a negative status. The results of this study, which was launched in 2023 and expected to run for 2 years, are eagerly awaited by the research community.

### 2.3 C5a receptor inhibitors

C5a is a protein fragment released from cleavage of complement component C5 by protease C5-convertase. C5a has been shown to upregulate the cell surface expression of PR3 and MPO, prompting neutrophils to respond to ANCA stimulation and cause tissue damage (Prendecki and McAdoo, 2021a). Neutrophils stimulated by ANCA can also activate the alternative complement pathway, further initiating, recruiting, and activating neutrophils in an inflammatory feedback loop. In addition to being expressed by neutrophils, the C5a receptor (C5aR) is expressed by other myeloid cells, including dendritic cells (DCs), eosinophils, and monocytes. The C5aR may also play a role in the pathogenesis of EGPA (Prendecki and McAdoo, 2021a).

Avacopan was the first oral C5aR inhibitor to be approved by the FDA. To date, avacopan has been approved in several countries, including the United States and Japan, for the treatment of AAV. The ADVOCATE study (Jayne et al., 2021) compared the efficacy, safety, and risk of infection associated with avacopan or a steroid tapering regimen in a cohort of 331 patients with AAV. The results showed that at 26 weeks post-treatment initiation, the clinical response of the avacopan group was no different from that of the prednisone reduction group; however, avacopan was superior to prednisone reduction in terms of sustained remission at 52 weeks. Moreover, avacopan performed better than prednisone reduction in terms of safety. Another study (Cortazar et al., 2023a) analyzing data from the ADVOCATE trial, suggested that after 52 weeks of treatment, the improvement in the estimated glomerular filtration rate (eGFR) was higher in the avacopan group than in the prednisone group among patients with severe renal impairment (eGFR ≤ 20 mL/min/1.73 m^2^). Cortazar et al. (2023b) reported three cases of rapidly progressive AAV requiring renal replacement therapy. These patients were treated with avacopan in combination with RTX and/or CYC, along with rapid steroid tapering. All patients showed significant improvement in renal function and successfully discontinued hemodialysis. Another RCT (Jayne et al., 2017) compared the control group treated with prednisone (60 mg/day, tapered to 0 mg within 20 weeks), against those treated with avacopan (30 mg/day) or low-dose prednisone (20 mg/day) plus avacopan (30 mg/day), using the main efficacy endpoint of a ≥ 50% reduction in Birmingham Vasculitis Activity Score at week 12. The results showed no inferiority in terms of safety and efficacy among the treatment groups. The study by Merkel et al. (2020) suggested that the addition of avacopan to the standard regimen of glucocorticoids combined with RTX or CYC was not only well tolerated, but also shortened the duration of remission at higher doses. Abe et al. (2023) reported four cases in which an interesting finding was observed; avacopan did not decrease ANCA titers while reducing AAV activity. van Leeuwen et al. (2022) reported that an 84-year-old patient with refractory AAV, who was successful treated avacopan, exhibited a progressive decrease in C3 levels. Thus, avacopan may improve the outcomes of refractory AAV patients with concomitant complement system activation. Based on current clinical evidence, the 2024 KIDGO guidelines (Kidney Disease: Improving Global Outcomes KDIGO ANCA Vasculitis Work Group, 2024) clearly recommend avacopan as an effective alternative therapy for patients who face increased toxicity risks from glucocorticoid treatment. These patients are likely to derive the greatest therapeutic benefit from avacopan therapy. Furthermore, the guidelines emphasize that avacopan treatment may be particularly helpful in promoting significant recovery of glomerular filtration rate (GFR) among patients with low GFR. Thus, avacopan provides clinicians with a new treatment strategy, facilitating the development of optimized therapeutic plans for patients and potentially improving their prognosis.

### 2.4 Anti-CD52 monoclonal antibody

Cellular immunity also plays a crucial role in the pathogenesis of AAV. CD4^+^ T-cells facilitate the production of ANCA, and both CD4^+^ and CD8^+^ T-cells recognize ANCA antigens deposited in peripheral tissues through activated neutrophils. Animal model studies have provided us with considerable insights into this process. The presence of T-cells has also been observed in the glomeruli and tubulointerstitium of AAV patients (Kitching et al., 2020; Prendecki and McAdoo, 2021b), further emphasizing the importance of cellular immunity in such diseases.

Alemtuzumab is a humanized anti-CD52 monoclonal antibody that depletes all lymphocytes and has a particularly long-lasting effect on T cells, resulting in a CD4^+^ T cell count that takes approximately 60 months to recover (Coles et al., 2006). The ALEVIATE study (Gopaluni et al., 2022) included 23 patients with refractory AAV or Behçet’s disease (BD). These patients were randomly assigned to receive either 60 mg or 30 mg of alemtuzumab. The study results showed that alemtuzumab provided relief to approximately 2/3 of the patients at 6 months. However, this relief was sustained in only 1/3 of the patients at 12 months. Additionally, there were no significant differences in clinical endpoints between the different dosage groups. Alemtuzumab, as an immunotherapy targeting T-cells, demonstrates some potential in the treatment of AAV. However, its long-term efficacy and optimal dosing strategy still require further research and optimization.

### 2.5 T cell costimulation modulators

T cells become activated when their T cell receptor interacts with a cognate antigenic peptide presented on a major histocompatibility complex molecule expressed on the surface of antigen-presenting cells. The role of T cell costimulatory molecules is to strengthen the extent of T cell activation. Given that T cells drive granuloma formation, T cell activation could be implicated in the pathogenesis of AAV (Wilde et al., 2011).

Abatacept is a CTLA-4-Ig fusion protein, which binds to the costimulatory ligands CD80 and CD86 and blocks their interaction with the costimulatory receptors CD28 and CTLA-4 expressed by T cells, thereby inhibiting T cell activation (Glatigny et al., 2019; Ortiz-Fernández et al., 2023). A real-world study (Mettler et al., 2022) showed that abatacept was effective in < 50% of patients with refractory and/or relapsing GPA; however, only six patients were included in the study. In another study (Langford et al., 2014) which included 20 patients with non-severe, relapsing GPA, 18 (90%) patients improved while 16 (80%) patients achieved remission following abatacept treatment. Moreover, prednisone could be discontinued in 11 of the 15 (73%) patients as a result of abatacept treatment. These findings indicate that abatacept is well tolerated in patients with non-severe relapsing GPA and achieves a high rate of disease remission, while permitting glucocorticoid discontinuation.

### 2.6 TNF⁃α inhibitor

Evidence (Kamesh et al., 2002) suggests that TNF-α plays a central role in the pathogenesis of AAV by activating neutrophils, which leads to vascular endothelial injury. Indeed, anti-TNF-α antibody treatment significantly reduced proteinuria, crescent formation, and the incidence of pulmonary hemorrhage in vasculitic mice (Little et al., 2006). These findings suggest that anti-TNF-α antibodies may be effective in treating diagnosed vasculitis.

Infliximab, etanercept, and adalimumab are anti-TNF-α monoclonal antibodies currently being considered as biologic therapies for AAV. A meta-analysis (Bala et al., 2020) of data from four RCTs suggested that etanercept did not show significant efficacy in achieving disease remission or preventing relapse in GPA patients (as well as a small subset of MPA patients). Although etanercept may have exerted a minimal or no impact on severe adverse effects, it could potentially increase the likelihood of adverse reactions, leading to treatment discontinuation. A 33-patient cohort study (Morgan et al., 2011) with a 12-month follow-up period concluded that infliximab in addition to standard treatment did not provide clinical benefits for patients with active AAV. Another multi-center, prospective study with a small sample (Booth A. et al., 2004) showed that induction therapy with infliximab resulted in remission in 88% of AAV patients and a reduction in their glucocorticoid dosage. A prospective study (Laurino et al., 2010), which included 14 patients with active AAV, found that using adalimumab in combination with CYC achieved remission in 11 (78.5%) patients within 14 weeks (mean 12 weeks); however, one patient died and three patients developed infections. Although the efficacy and safety of this regimen were similar to those of the standard treatment, it did permit a reduction in the level of glucocorticoid exposure. Lamprecht et al. (2002) used infliximab for induction therapy in six patients with refractory GPA. The results showed that five patients achieved remission, with a gradual reduction in glucocorticoid dosage; furthermore, these patients remained in remission during the 6–24 months of follow-up. Kleinert et al. (2004) have reported a case of a patient with severe orbital Wegener granuloma who experienced acute renal failure despite receiving aggressive conventional immunosuppressive therapy. However, following the initial infusion of infliximab, there was an improvement in the patient’s renal function, which persisted throughout the course of treatment. The results of a multicenter cohort study (Silva et al., 2011), which included 153 patients with GPA accompanied by vasculitis, revealed that all 13 cases of newly diagnosed solid malignancies were associated with CYC exposure. Specifically, eight cases occurred in the group receiving etanercept, while five cases arose in the placebo group. Moreover, the etanercept group had a significant increased risk of solid malignancy compared with that of the general population. Thus, etanercept treatment appears to increase the risk of malignancy in patients receiving cytotoxic drugs.

### 2.7 Anti-IL-6-receptor (IL-6R) antibody

IL-6 is a notorious B lineage differentiation factor, which promotes the *in situ* activation of macrophages, the differentiation of T lymphocytes, and the synthesis of other proinflammatory cytokines (Naka et al., 2002). Inflammatory cytokines and chemokines may play a role in the pathogenesis of AAV (Chen and Kallenberg, 2010). Indeed, some studies have reported raised serum IL-6 levels in AAV patients versus healthy controls (Booth AD. et al., 2004; Krajewska Wojciechowska et al., 2019).

Tocilizumab, an anti-IL-6R antibody, has shown good clinical benefits in the biological treatment of patients with giant cell arteritis (Antonio et al., 2022). Tang et al. (2023) reported findings from a patient with refractory GPA and elevated IL-6 expression. After treatment with tocilizumab, the patient’s symptoms improved and the levels of inflammatory markers, including IL-6, normalized. The study by Berti et al. (2015) found that serum IL-6 levels were significantly increased in AAV patients; moreover, the IL-6 was predominantly expressed at sites of active vasculitis. Crucially, tocilizumab treatment induced complete and sustained disease remission in patients with severe multisystem MPA. In a prospective, single-center cohort study (Sakai et al., 2016) of tocilizumab monotherapy in MPA, two of six patients (33.3%) achieved complete remission at 6 months and three patients (50.0%) achieved complete remission at 12 months. Four patients (66.7%) discontinued treatment after 1 year and were relapse-free for 6–15 months at the time of their last follow-up visit. In view of this, it was concluded that tocilizumab monotherapy is a feasible treatment strategy for some MPA patients.

### 2.8 Anti-IL-5/IL-5-receptor (IL-5R) antibody

IL-5, a cytokine mainly involved in the chemotaxis and activation of eosinophils, is also involved in the pathogenesis of EGPA; thus, targeting IL-5 or its receptor represent promising EGPA treatment strategies (Furuta et al., 2019; Vega Villanueva and Espinoza, 2020).

Mepolizumab is currently the representative drug of anti-IL-5 antibodies, while the representative drugs of IL-5R antibodies are benralizumab and reslizumab. In 2023, Europe published the first evidence-based guidelines for the diagnosis and treatment of EGPA (Emmi et al., 2023). The report summarized a number of important clinical studies (Kahn et al., 2010; Kim et al., 2010; Moosig et al., 2011; Wechsler et al., 2017; Steinfeld et al., 2019; Bettiol et al., 2022; Hadjadj et al., 2022) and recommended that the combination of mepolizumab and glucocorticoids is used to induce remission in patients with relapsed and refractory EGPA without organ damage or other life-threatening complications. Meanwhile, RTX, mepolizumab, or conventional disease-modifying anti-rheumatic drugs combined with glucocorticoids are recommended for maintaining remission in patients with severe EGPA. For patients with non-severe EGPA, glucocorticoids alone or in combination with mepolizumab are currently recommended. Meanwhile, patients with non-severe EGPA and recurrent respiratory symptoms are advised to increase their glucocorticoid dose and/or take mepolizumab. The latest single-center study in Japan (Yamane and Hashiramoto, 2023) also confirmed the efficacy of mepolizumab, as evidenced by its ability to induce remission in EGPA patients treated with glucocorticoids. After 3 years of mepolizumab treatment, glucocorticoids could be discontinued in approximately 50% of EGPA patients, even in those with severe or ANCA-positive EGPA. The 2023 European guidelines (Emmi et al., 2023) suggest that other IL-5 or IL-5R inhibitors, such as benralizumab and relizumab, may be considered in patients refractory to mepolizumab, as their efficacy has been reported in case reports and case series (Koga et al., 2022; Koike et al., 2023). However, the latest retrospective study (Cottu et al., 2023) suggests that benralizumab is an effective treatment for EGPA with refractory asthma or respiratory symptoms in its own right, and can help reduce the dosage of glucocorticoids. By contrast, the efficacy of mepolizumab was lower in patients who had previously failed benralizumab therapy. Another retrospective study (Nanzer et al., 2024) suggests that among 70 EGPA patients treated with benralizumab, 47 patients (67.1%) achieved clinical remission at 1 year, with a similar remission rate maintained at 2 years. 87.1% of patients were relapse-free at 1 year, and among the 53 patients who completed 2 years of treatment, there was an 84.9% relapse-free rate. A total of 67.9% of patients no longer required any glucocorticoids to control their disease. The latest Italian real-world study (Nolasco et al., 2023) discussed the efficacy and safety of mepolizumab and benralizumab in patients with refractory EGPA treated for 24 months. The results suggest that mepolizumab and benralizumab are effective and safe as long-term add-on therapies for patients with EGPA. In 2024, a multicenter, double-blind, phase 3, randomized trial (Wechsler et al., 2024) was conducted involving 140 patients with relapsing or refractory EGPA. The study compared the efficacy and safety of benralizumab versus mepolizumab. The results indicated that benralizumab was non-inferior to mepolizumab in inducing remission among patients with relapsing or refractory EGPA and demonstrated a better safety profile.

## 3 Conclusion

Finally, this article summarizes the key and latest clinical studies in the form of tables for readers’ convenience (Tables 1, 2). The rapid development of biotherapy has played an important role in both AAV-induced remission and maintenance. Biologic research provides new avenues for improving the prognosis of AAV patients and reducing adverse reactions associated with many other forms of immunotherapy. Experts recommend RTX as a first-line treatment option for AAV, for the induction of remission, maintenance therapy, or the treatment of relapsed patients. Avacopan has also received considerable attention in the treatment of AAV by significantly reducing glucocorticoid dosage and adverse reactions without affecting therapeutic efficacy. The sequential use of belimumab and RTX has demonstrated promise in controlling the recurrence of AAV and is likely to become a research hotspot in the future. Phase II studies suggest that alemtuzumab demonstrates some efficacy in the treatment of refractory AAV, but the recurrence rate is high. Abatacept has proved effective in patients with non-severe, recurrent GPA, while facilitating glucocorticoid discontinuation; however, these conclusion are currently based on low-quality data. To date, inhibiting TNF⁃α has not proved effective as a treatment for AAV, and the safety of this method also remains a concern. On the basis of reports that serum IL-6 levels are increased in AAV patients, small studies and case reports have shown that tocilizumab significantly increases complete and sustained remission rates. Meanwhile, mepolizumab has demonstrated efficacy in the treatment of EGPA and is especially recommended for patients with recurrent and refractory EGPA. Belimumab can be used as a second-line regimen for patients with mepolizumab resistance. The second-generation anti-CD20 antibody ofatumumab has demonstrated good efficacy in a series of reports (McAdoo et al., 2016). The third-generation anti-CD20 antibody obinutuzumab has shown stronger effects in B-cell reduction and NK cell activation (Urlaub et al., 2019). Eculizumab is a molecule that blocks the cleavage of C5 complement components into C5a and C5b, and there are currently only successful case reports available (Ribes et al., 2019). As there is not enough clinical evidence for these drugs at present, this article does not provide further discussion. We should look forward to the clinical results of these new biologics. In conclusion, biologics offer new hope to AAV patients, by improving therapeutic efficacy and alleviating adverse reactions typically associated with immunotherapy. However, the development of biologics for AAV is still in its early stages, and more basic research and clinical data are needed to verify their safety and efficacy, especially in the long-term. Thus, the optimization of biologics will be a major focus of future AAV research.

**TABLE 1:** Key clinical study of biologics for GPA and/or MPA.

Study	Design(n)	Population	Intervention	Main conclusion
RAVE Stone et al. (2010): (NCT00104299)	Multicenter, randomized, double-blind, double-dummy, noninferiority trial. (*n* = 197)	GPA or MPA newly diagnosed or relapsing, SCr <353 μmol/L	RTX/AZA versus CYC/AZA	RTX noinferior to CYC; RTX may be better for relapsing AAV
RITUXVAS Jones et al. (2015); Jones et al. (2010a): (ISRCTN28528813)	International, randomised, controlled, prospective, open trial. (*n* = 44)	GPA or MPA newly diagnosed, renal involvement	CYC + RTX versus CYC/AZA	At 12 months, a RTX-based regimen was not superior to standard intravenous CYC. At 24 months, rates of the composite outcome of death, end-stage renal disease and relapse did not differ between groups. In the RTX group, B cell return was associated with relapse
MAINRITSAN Guillevin et al. (2014); Terrier et al. (2018): (NCT00748644)	Non-blind randomized controlled trial. (*n* = 115)	GPA or MPA in remission after CYC and GCs	RTX (500 mg, every 6 months) versus AZA	Remission rates for RTX were higher at 28 months. Similar rates of adverse events. Decreased relapse rate at long-term follow-up
MAINRITSAN2 Charles et al. (2018): (NCT01731561)	Open-label, multicentre, randomised controlled trial. (*n* = 162)	GPA or MPA in remission	Scheduled RTX versus RTX tailored to B cell return and/or ANCA	No difference in relapse rates. Tailored RTX arm received fewer infusions
MAINRITSAN3 Charles et al. (2020): (NCT02433522)	Multicenter, randomized controlled trial. (*n* = 97)	GPA or MPA, sustained remission, 2 years after RTX maintenance therapy	Placebo versus 2 further years of RTX	Compared to the placebo group, extending the course of RTX treatment reduces the recurrence rate
RITAZAREM Gopaluni et al. (2017); Smith et al. (2020, 2023): (NCT01697267)	International, multicenter, open-label, randomized controlled trial. (*n* = 188)	Relapsed GPA or MPA re-induced with RTX and GCs, in remission	RTX (1 g every 4 months) versus AZA	RTX superior in preventing relapse. no differences in rates of hypogammaglobulinaemia or infection between groups
Morel et al. (2022)	Retrospective, multicentre study. (*n* = 153)	Serum creatinine level ≥350 μmol/L and/or eGFR ≤ 15 mL/min/1.73 m^2^	RTX versus CYC and CYC versus CYC + PE	NO difference in efficacy between RTX and CYC.PE has short-term benefit
Ishikawa et al. (2023)	Real world research. (*n* = 687)	Life-threatening AAV	RTX versus CYC	RTX similar short-term effectiveness on mortality to CYC, RTX short-term renal prognosis inferior to CYC
Delestre et al. (2023)	Multicenter, prospective study. (*n* = 687)	GPA or MPA in remission	AZA, 18-month fixed-schedule RTX, 18-month tailored RTX and 36-month RTX	84-month remission rate is higher with an 18-month fixed RTX regimen. 36-month RTX does not reduce the long-term relapse rate
LoVAS Furuta et al. (2021, 2023): (NCT02198248)	Phase 4, multicenter, open-label, randomized, noninferiority trial. (*n* = 140)	New-onset AAV without severe glomerulonephritis or alveolar haemorrhage	Reduced-dose GCs plus RTX or high-dose GCs plus RTX	Reduced-dose group noinferior to high-dose group. At 24 months no difference in relapse rates. SAEs were less frequent in the reduced-dose group
Thomas et al. (2021)	Multicenter, observational, retrospective study. (*n* = 162)	GPA or MPA	Induction: RTX versus CYC. Maintenance: RTX versus other agents	The SI incidence was higher during CYC compared to RTX induction while there was no difference between RTX and other agents used for maintenance therapy
RaVeR Merkel et al. (2021): (NCT01613599)	Phase IV, open-label, prospective study. (*n* = 97)	GPA or MPA	Patients initiating treatment with rituximab were evaluated every 6 months for up to 4 years	The safety profile of long-term treatment with RTX remains consistent
Thietart et al. (2022)	Multicenter cohort study. (*n* = 93)	GPA or MPA≥75 years old	Induction: high-dose GCs plus RTX verus RTX. Maintenance: RTX	RTX was associated with achievement and maintenance of remission. The incidence of SI and death was high when RTX plus high-dose GCs was used as induction therapy but not when RTX was used as maintenance therapy
BREVAS Jayne et al. (2019): (NCT01663623)	Multicenter, double-blind, placebo-controlled study. (*n* = 105)	GPA or MPA after induced remission	Belimumab plus AZA and low-dose oral GCs versus placebo	Belimumab plus AZA and GCs for the maintenance of remission in AAV did not reduce the risk of relapse
COMBIVAS McClure et al. (2023): (NCT03967925)	Randomised, double-blind, placebo-controlled trial	AAV with active disease, PR3 ANCA+	RTX + GCs plus belimumab versus plus placebo	Primary endpoint of this study is time to PR3 ANCA negativity
ADVOCATE Jayne et al. (2021); Cortazar et al. (2023a): (NCT02994927)	Randomized, controlled trial. (*n* = 331)	GPA or MPA received either CYC (followed by AZA) or RTX	Avacopan versus oral GCs on a tapering schedule	Avacopan was noninferior but not superior to GCs taper with respect to remission at week 26 and was superior to GCs taper with respect to sustained remission at week 52. Among patients with baseline eGFR ≤20 mL eGFR improved more in the avacopan group
CLEAR Jayne et al. (2017): (NCT01363388)	Randomized, placebo-controlled trial. (*n* = 67)	GPA or MPA received either CYC or RTX	Placebo plus high-dose GCs; avacopan plus reduced-dose GCs; avacopan	There was no significant difference in efficacy or adverse events
ALEVIATE Gopaluni et al. (2022): (NCT01405807)	Randomised, prospective, open-label, dose ranging clinical trial. (*n* = 23)	Refractory AAV or Behcets treatment	60 mg or 30 mg alemtuzumab	At 6 months, 2/3 of patients experienced disease remission. An additional 1/3 of patients achieved remission by 12 months. 30% of patients experienced serious adverse events, with 4 cases specifically associated with alemtuzumab
Mettler et al. (2022)	Retrospective study. (*n* = 26)	Refractory and/or relapsing GPA	Infliximab, adalimumab or abatacept	Efficacy in less than 50%
Langford et al. (2014)	Open-label prospective trial. (*n* = 20)	Non-severe relapse of GPA	All eligible patients were treated with abatacept on days 1, 15, 29 and every 4 weeks thereafter	Abatacept was well tolerated and was associated with a high frequency of disease remission and prednisone discontinuation
Bala et al. (2020) (NCT01275287)	Systematic reviews. (*n* = 440)	GPA, MPA or EGPA	Mepolizumab versus placebo; belimumab or etanercept versus placebo; infliximab versus rituximab	Mepolizumab may reduce relapse rates in patients with relapsing or refractory EGPA. Etanercept or belimumab use in GPA or MPA patients may increase withdrawal risk due to AEs and have minimal impact on SAEs. Etanercept may not significantly aid in durable remission
Booth et al. (2004a)	Open-label, multi-center, prospective clinical. (*n* = 32)	Acute AAV; persistent AAV	Infliximab plus GCs and CYC; infliximab plus GCs tapered according to clinical status	Induction therapy with infliximab resulted in remission in 88% of AAV patients and a reduction in their GCs dosage
Laurino et al. (2010)	Phase II, open-label, prospective study. (*n* = 14)	GPA or MPA newly diagnosed or relapsing	Adalimumab plus CYC and a reducing course of GCs	The efficacy and safety of this regimen were similar to those of the standard treatment
Silva et al. (2011)	Multicenter longitudinal cohort. (*n* = 153)	GPA	Etanercept or placebo in addition to standard therapy	Etanercept treatment appears to increase the risk of malignancy in patients receiving cytotoxic drugs
Sakai et al. (2016)	Prospective single-arm, single-center, cohort, pilot study. (*n* = 6)	New-onset MPA	Tocilizumab monotherapy	Tocilizumab monotherapy is a feasible treatment strategy for some MPA patients

ANCA, anti-neutrophil cytoplasmic antibody; GPA, granulomatosis with polyangiitis; MPA, microscopic polyangiitis; AZA, azathioprine; CYC, cyclophosphamide; GCs, glucocorticoids; RTX, rituximab; SCr, serum creatinine; CYC/AZA, After induction of remission with CYC, treatment was maintained with AZA; RTX/AZA, After induction of remission with RTX, treatment was maintained with AZA; PE, plasma exchange; AEs, Adverse events; SAEs, serious adverse events; SI, serious infections.

**TABLE 2:** Key clinical study of biologics for EGPA.

Study	Design(n)	Population	Intervention	Main conclusion
Bettiol et al. (2022)	Multicenter, retrospective study. (*n* = 203)	EGPA	100 mg or 300 mg mepolizumab	Mepolizumab at both 100 mg every 4 weeks and 300 mg every 4 weeks is effective
Canzian et al. (2021)	Retrospective European collaborative study. (*n* = 147)	Refractory and/or relapsing EGPA	RTX; mepolizumab; omalizumab	RTX may effectively treat EGPA vasculitis relapses, while mepolizumab is highly effective and safe for GC-dependent asthma patients
MIRRA Wechsler et al. (2017); Steinfeld et al. (2019): (NCT02020889)	Randomized, placebo-controlled, double-blind, parallel-group, phase 3 trial. (*n* = 136)	Refractory and/or relapsing EGPA	300 mg mepolizumab versus placebo	Mepolizumab led to more weeks and a higher rate of participants in remission. Post hoc analysis suggests >75% of patients derived benefit
Yamane and Hashiramoto (2023)	Single-center retrospective study. (*n* = 27)	EGPA receiving induction therapy with GCs and mepolizumab	GC-free group versus GC-continue group	After 3 years of mepolizumab, about 50% of EGPA patients, even severe or ANCA-positive ones, could stop GCs
Cottu et al. (2023)	Multicentre, retrospective study. (*n* = 68)	Refractory EGPA	Benralizumab was added to the original regimen	Benralizumab effectively treats EGPA with refractory asthma or respiratory symptoms, reducing GCs dosage. Conversely, mepolizumab’s efficacy was lower in patients unsuccessful with benralizumab
Nanzer et al. (2024)	Retrospective cohort. (*n* = 70)	EGPA	GCs plus benralizumab	Benralizumab treatment was well-tolerated, leading to GC-free remission for most patients
Nolasco et al. (2023)	Multicenter observational study. (*n* = 49)	Refractory and/or relapsing EGPA	Benralizumab; mepolizumab	Mepolizumab and benralizumab are effective and safe as long-term add-on therapies
MANDARA Wechsler et al. (2024): (NCT02020889)	Multicenter, double-blind, phase 3, randomized, active-controlled noninferiority trial. (*n* = 140)	Refractory and/or relapsing EGPA	Benralizumab versus mepolizumab	Benralizumab was noninferior to mepolizumab for the induction of remission

ANCA, anti-neutrophil cytoplasmic antibody; GPA, granulomatosis with polyangiitis; MPA, microscopic polyangiitis; CYC, cyclophosphamide; GCs, glucocorticoids; RTX, rituximab.
